# Mitochondrial transcription factor A (TFAM) shapes metabolic and invasion gene signatures in melanoma

**DOI:** 10.1038/s41598-018-31170-6

**Published:** 2018-09-21

**Authors:** L. F. Araujo, A. D. D. Siena, J. R. Plaça, D. B. Brotto, I. I. Barros, B. R. Muys, C. A. O. Biagi, K. C. Peronni, J. F. Sousa, G. A. Molfetta, L. C. West, A. P. West, A. M. Leopoldino, E. M. Espreafico, W. A. Silva

**Affiliations:** 1 Department of Genetics-Ribeirão Preto Medical School, University of São Paulo, Ribeirão Preto, Brazil; 2 National institute of Science and Technology in Stem Cell and Cell Therapy, Center for Cell-based Therapy-CEPID/FAPESP, Ribeirão Preto, Brazil; 3 Department of Cellular and Molecular Biology-Ribeirão Preto Medical School, University of São Paulo, Ribeirão Preto, Brazil; 4 Department of Clinical Analysis-Toxicology and Food Sciences, School of Pharmaceutical Sciences of Ribeirão Preto, University of São Paulo, Ribeirão Preto, Brazil; 5 Microbial Pathogenesis & Immunology, Health Science Center, Texas A&M University, College Station, USA; 6 Center for Integrative System Biology-CISBi-NAP/USP, University of São Paulo, Ribeirão Preto, Brazil; 7 Medical Genomics Laboratory, CIPE, AC Camargo Cancer Center, São Paulo, Brazil

## Abstract

Mitochondria are central key players in cell metabolism, and mitochondrial DNA (mtDNA) instability has been linked to metabolic changes that contribute to tumorigenesis and to increased expression of pro-tumorigenic genes. Here, we use melanoma cell lines and metastatic melanoma tumors to evaluate the effect of mtDNA alterations and the expression of the mtDNA packaging factor, TFAM, on energetic metabolism and pro-tumorigenic nuclear gene expression changes. We report a positive correlation between mtDNA copy number, glucose consumption, and ATP production in melanoma cell lines. Gene expression analysis reveals a down-regulation of glycolytic enzymes in cell lines and an up-regulation of amino acid metabolism enzymes in melanoma tumors, suggesting that TFAM may shift melanoma fuel utilization from glycolysis towards amino acid metabolism, especially glutamine. Indeed, proliferation assays reveal that TFAM-down melanoma cell lines display a growth arrest in glutamine-free media, emphasizing that these cells rely more on glutamine metabolism than glycolysis. Finally, our data indicate that TFAM correlates to VEGF expression and may contribute to tumorigenesis by triggering a more invasive gene expression signature. Our findings contribute to the understanding of how TFAM affects melanoma cell metabolism, and they provide new insight into the mechanisms by which TFAM and mtDNA copy number influence melanoma tumorigenesis.

## Introduction

Melanoma is a malignancy caused by a stochastic process model of mutation events in melanocytes, pigment-producing cells that can be found in the skin throughout the body and other organs^1^. Melanoma follows a typical progression, categorized based on cellular penetration: radial growth phase (RGP), vertical growth phase (VGP) and metastatic melanoma (MET). Although histologically these stages are well characterized, molecular approaches are crucial to predict survival and to guide therapy^1^.

In melanoma, the most mutated driver genes (BRAF, RAS, and NF1) code for members of the MAPK pathway, a canonical signalling pathway that transfers mitogenic signals from growth factors to the nucleus through the activation of Ras GTPase and RAF/MEK/ERK kinases^2^. Although these gene products act on the same pathway, each mutated subtype has its own gene expression profile^2^. Also, the most mutated of these genes in melanoma is BRAF. Approximately 52% of all melanomas harbor a valine to glutamic acid substitution (V600E), which causes constitutive kinase activation^3,4^.

The BRAF^V600E^ mutation has been reported to regulate energetic metabolism of melanoma cells via mitochondrial biogenesis. Haq *et al*. showed that BRAF-mutated cells exhibit downregulation of oxidative enzymes, a reduction of mitochondrial function, and increased lactate production^5^. Altered energetic metabolism is known as a hallmark of cancer and is necessary to supply the metabolic needs of highly proliferative cells^6^. Metabolic reprogramming in cancer, first described by Otto Warburg, describes the process by which, independent of the oxygen conditions, cancer cells switch their metabolism from oxidative phosphorylation (OXPHOS) to aerobic glycolysis, resulting in the conversion of pyruvate to lactate rather than oxidation within mitochondria^7^. To compensate for the low efficiency in ATP production of aerobic glycolysis, many cancer cells increase glucose uptake.

In this context, many oncogenes regulate energetic metabolism^8^, including other metabolic pathways such as glutamine metabolism. An increased uptake of glutamine was previously observed in highly proliferative cells, and its metabolism was used to feed nucleotide and amino acid biosynthetic pathways^9^. Additionally, it has been reported that cancer cells with mitochondrial defects rely on glutamine metabolism to replenish tricarboxylic acid cycle intermediates (anaplerosis) essential for maintaining the function of the major biosynthesis pathways^10^.

Due to the central role for mitochondria in metabolism, many studies have investigated how changes in mitochondrial DNA (mtDNA) affect mitochondrial function and cancer metabolism. MtDNA depletion^11^, mutations^12,13^, OXPHOS impairment, altered mitochondrial reactive oxygen species (ROS) production and metabolite imbalances can trigger mitochondrial dysfunction, leading to mitochondrial stress signalling^14,15^ that can promote the expression of nuclear genes contributing to tumorigenesis^16,17^. Moreover, mitochondrial dysfunction has been reported to promote tumor growth via silencing of TFAM^18,19^, a nuclear-encoded mitochondrial DNA packaging and transcription factor that is responsible for mtDNA maintenance^20^. TFAM homozygous knockout mice displayed non-functional OXPHOS and severe mtDNA depletion, resulting in embryonic lethality, whereas TFAM heterozygous knockout manifested reduction in mtDNA copy number (mtDNAcn) and OXPHOS deficiency in cardiac tissue^21^.

In this study, we performed a correlation analysis of mtDNA content, mutational load, and TFAM expression with the energetic metabolism of melanoma cell lines. We observed a positive correlation between mtDNA content and both glucose consumption and ATP production. We then expanded our data using melanoma cell lines and metastatic melanoma samples, and observed that TFAM mediates the expression of metabolic genes, demonstrating a role for this mitochondrial transcription factor in metabolic reprogramming. Our data also suggest an additional pro-tumorigenic role of TFAM, as we show increasing the expression of angiogenesis and invasion genes.

## Results

### Evaluation of mitochondrial genome alterations in melanoma cell lines

To evaluate mitochondrial genome alterations, we sequenced the mitochondrial genome and quantified mtDNA content of 9 melanoma cell lines (WM35, WM1552C, WM1789, WM278, WM902, WM793, 1205 LU, WM1617 and WM9) and one melanocyte (FM308). In sequencing, we achieved an average coverage of 55x per genome. To minimize errors, we only considered variants supported by at least nine reads^22^. Mitochondrial genome sequences from each cell line were compared to the revised human Cambridge reference sequence (rCRS)^23^, as most of cell lines in our study were derived from patients with no non-neoplastic control available.

**Figure 1:**
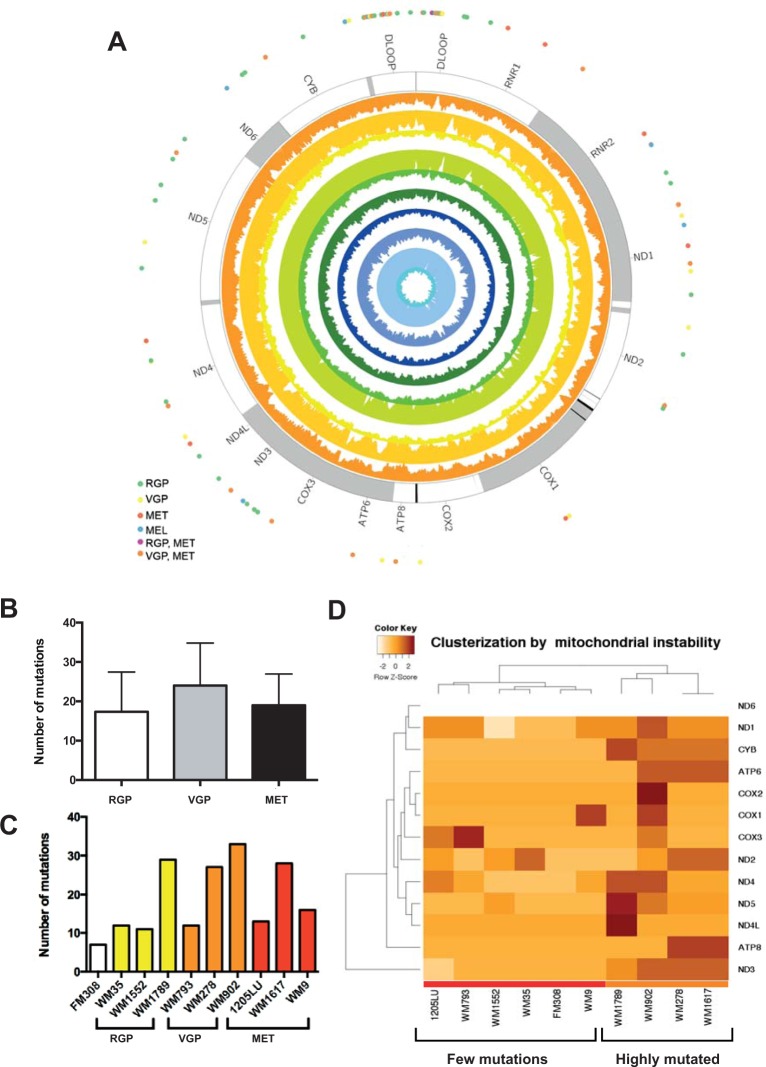
mtDNA mutational analysis in primary melanocytes and melanoma cell lines. (**A**) Circos plot of the mtDNA. External dots represent all the 86 mutation events in corresponding genes, and inner circular plots represent the average coverage of the sequencing for each cell line in the following order (from inside): FM308, WM35, WM1552, WM1789, WM278, WM793, WM902, 1205Lu, WM1617 and WM9; (**B**) There was no difference in the mtDNA mutation load among the melanoma stages (p = 0.6969, Kruskal-Wallis test); (**C**) Total number of mtDNA mutations per cell line. Note that mutation rates varied independently of the melanoma stage; (**D**) Unsupervised hierarquical clustarization analysis using the frequency of mutations in protein-coding genes. The clustarization was able to distinguish two mutated cell lines clusters (color bar at the bottom). Red color bar shows the cell line cluster with few mutations and the orange color bar the mtDNA highly mutated cluster. ND6 row is blank because the ND6 gene was hit by only one synonymous mutations in all cell lines, therefore there was not variation among the cell lines (Supplementary Table S1). Note in C, that genetically paired cell lines (WM793/1205Lu and WM278/WM1617) consistently showed equal number of mutations between the members of each pair. RGP: Radial Growth Phase; VGP: Vertical Growth Phase; MET: Metastatic Melanoma.

We found a total of 86 variants, from which 47% (40/86) were in non-protein coding regions, such as DLOOP, tRNA and rRNA, and 53% (46/86) of them affected protein coding genes. Separating the coding mutations by protein effect, 34% (29/86) of the total were synonymous and 19% (17/86) were non-synonymous (see Supplementary Table S1) (Fig. 1A).

Our cell lines represented individual melanoma stages, allowing us to evaluate mutational load by stage. However, there was no difference in mutation accumulation in different stages (p = 0.6969, Kruskall-Wallis test) (Fig. 1B). Additionally, mutational load was very heterogeneous across the cell lines (Fig. 1C).

To establish a mutational pattern, we performed an unsupervised hierarchical clusterization using the mutation frequencies of mitochondrial protein coding genes of each melanoma cell line. As a result, we found two differently mutated clusters, one cluster encompassing five melanoma cell lines with few mutations (red colour bar at the bottom of Fig. 1D) and another with 4 melanoma cell lines harbouring many mtDNA mutations (orange colour bar, Fig. 1D). The cell lines grouped independently of melanoma staging (Fig. 1D).

**Figure 2:**
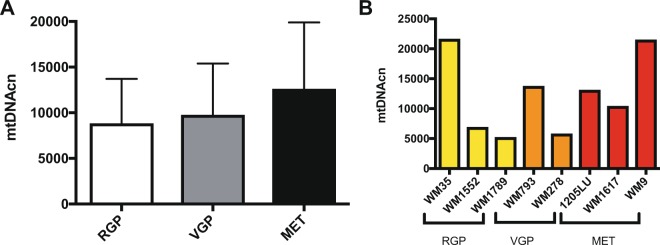
mtDNA copy number analysis in the melanoma cell lines. (**A**) Average mtDNA content of melanoma cell lines per staging (RGP, VGP and metastatic stages) (p = 0.9873, Kruskal-Wallis test); (**B**) Average mtDNA copy number for each cell line. RGP: Radial Growth Phase; VGP: Vertical Growth Phase; MET: Metastatic Melanoma; mtDNAcn: Mitochondrial DNA copy number.

We performed a similar analysis with mtDNAcn. As we observed in the mutation analysis, there was no difference in the mtDNA content among the melanoma stages (p = 0.6024, Kruskall-Wallis test) (Fig. 2A) and the mtDNA content also varied among the cell lines (Fig. 2B). Interestingly, two of the cell lines with the lowest mtDNAcn (WM1789 and WM278) also are in the high mutation load group. The other two highly mutated cell lines (W902 and W1617) have intermediate mtDNAcn and the cell lines with the highest mtDNAcn (WM9 and WM35) are in the few mutation group, indicating a possible negative-correlation between mtDNAcn and mutation accumulation.

Briefly, these data indicate no difference in the mutational load and mtDNA content in cell lines representing different stages of melanoma progression. However, in our analysis each staging group only had three cell lines, is insufficient to draw any conclusion. Therefore, we proceeded to evaluate the cell lines independently of melanoma staging.

### Impact of mtDNA alterations and TFAM expression on energetic metabolism of melanoma cell lines

We next evaluated if mtDNA alterations were associated with energetic metabolism in eight melanoma cell lines (WM35, WM1552C, WM1789, WM278, WM793, 1205 LU, WM1617 and WM9). To do so, we correlated mtDNA high/few mutations clusters (Colour bar at Fig. 1D) and mtDNAcn (Fig. 2B) with hydrogen peroxide production, ATP levels, glucose consumption and lactate production.

**Figure 3:**
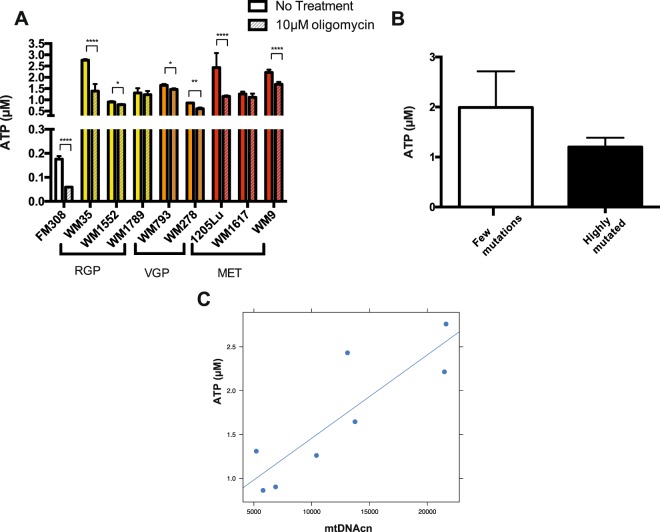
Rates of ATP content in the melanocyte and melanoma cell lines. (**A**) ATP basal levels in the melanocyte and melanoma cell lines. Oligomycin treatment was used as a control of the reaction. (**B**) Average basal levels of ATP in the two groups of cell lines clustered according to mtDNA mutation clusters (p = 0.06, Student t test); (**C**) Correlation between ATP levels and mtDNAcn (r = 0.86, p = 0.002, Pearson Correlation).RGP: Radial Growth Phase; VGP: Vertical Growth Phase; MET: Metastatic Melanoma; mtDNAcn: Mitochondrial DNA copy number. ATP measurements were performed in the following melanoma cell lines: WM35, WM1552, WM1789, WM793, WM278, 1205Lu, WM1617 and WM9.

Interestingly, ATP levels, which were equivalent among melanoma cells, were shown to be 5- to 10-fold higher in the melanoma cells than in primary melanocytes (Fig. 3A). Also, ATP levels were less influenced by oligomycin treatment in melanoma cells than in melanocytes (Fig. 3A), which might be expected given that melanoma cells rely less on OXPHOS than non-transformed melanocytes.

**Figure 4:**
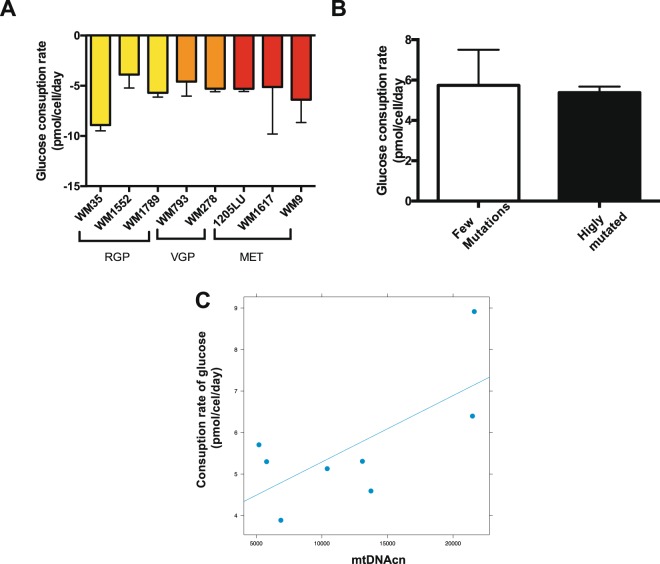
Glucose consumption rates in melanoma cell lines. (**A**) Glucose consuption rates (qGlc) for the indicated melanoma cell lines. The negative number indicates consuption of the metabolite over a period of 24 hours. (**B**) Average glucose consuption rates for the mtDNA mutated clusters of cell lines (p = 0.7480, Student t test); (**C**) Positive correlation between qGlc and mtDNAcn (r = 0.693, p = 0.0282, Pearson Correlation); For the B and C graphs we ploted the absolute number of qGlc. RGP: Radial Growth Phase; VGP: Vertical Growth Phase; MET: Metastatic Melanoma; mtDNAcn: Mitochondrial DNA copy number. Glucose consumption rates were evaluated in the following melanoma cell lines: WM35, WM1552, WM1789, WM793, WM278, 1205Lu, WM1617 and WM9.

Next, correlation analysis showed a positive correlation of mtDNAcn with ATP content (r = 0.86, p = 0.002, Pearson Correlation) (Fig. 3C). Additionally, glucose consumption (Fig. 4A) was also positively correlated with mtDNAcn (r = 0.693, p = 0.0282, Pearson Correlation) (Fig. 4C). These results suggest that melanoma cell lines with low mtDNAcn consume less glucose and produce lower amounts of ATP than those with higher mtDNAcn. We did not find any association of either ATP levels or glucose consumption with the differentially mutated clusters (Figs 3B and 4B). Also, no association was found between any mtDNA parameters with hydrogen peroxide levels (Supplementary Fig. S1) or lactate production (Supplementary Fig. S2). As TFAM is known as mtDNAcn regulator, we also compared these energetic parameters with TFAM expression. For the TFAM expression analysis, the following melanoma cell lines were evaluated: WM35, WM1552C, WM1789, WM278, WM793, 1205 LU and WM1617. However due to the limited number of cell lines, the results did not reach statistical significance (Supplementary Fig. S3). Nevertheless, these data suggest that mtDNA content in melanoma seems to affect metabolic features, such as glucose consumption and ATP production. To further evaluate signalling changes that might explain this correlation, we expanded our cell line panel using publicly available datasets^24^.

### TFAM is associated with nuclear gene expression alterations in melanoma cell lines

Aiming to further investigate if TFAM/mtDNAcn is related to energetic metabolism, we performed an RNA-seq analysis in seven melanoma cell lines (WM35, WM1552C, WM1789, WM278, WM793, 1205 LU and WM1617), which we have previously evaluated for metabolic parameters, searching for gene expression alterations that could explain the correlation of mtDNAcn with glucose consumption and ATP production.

In order to increase our sample size, we expanded our analysis joining transcriptomic data generated in our laboratory with public datasets. For that, we used RNA-seq data previously generated by Pawlikowski *et al*.^24^. Our expanded panel showed that melanoma cell lines were very heterogeneous in terms of TFAM expression (Fig. S4A). Unfortunately, we were not able to evaluate expression changes triggered by alterations in mtDNA content upon expansion of the panel, since raw DNA sequencing data was not available. As positive correlation between mtDNAcn and TFAM is well described in the literature^11,25^, we focused solely on the impact of TFAM on gene expression of melanoma cell lines.

**Figure 5:**
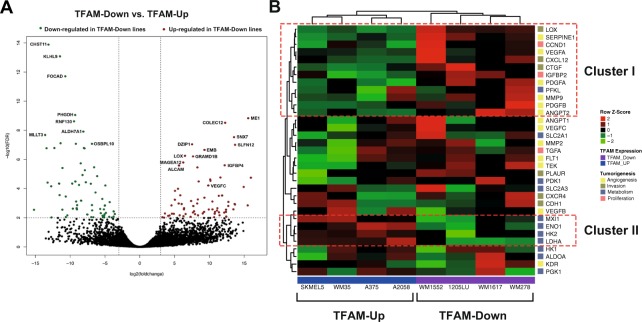
Differential expression analysis in melanoma cell lines with different TFAM expression, joining transcriptomic data generated by our group and by Pawlikowski *et al*.^24^. (**A**) Volcano plot showing the highly different expressed genes up (red dots) and down-regulated (green dots) in TFAM down cell lines; (**B**) Heatmap with the gene expression pattern of the HIF-α main targets, that were categorazed according with their role in tumorigenesis. The analysis revealed two distinct genes clusters, highlighted in dotted red lines. FDR: False discovery rate.

Using our expanded panel, we selected two groups of cell lines with different TFAM expression: TFAM Up and TFAM Down (Fig. S4B). All the melanoma cell lines included the BRAF^V600E^ mutation. Differential expression analysis between the two groups of melanoma cell lines showed 295 differently expressed genes (DEGs) (FDR < 0.05), from which 131 were down-regulated and 164 were up-regulated in TFAM down cell lines (Fig. 5A and Supplementary Table S2 - Tab DEGs WM melanoma cell lines), indicating clear gene expression differences between the groups. Interestingly, among the up-regulated genes in TFAM down cell lines, we found two targets of HIF1-α: LOX and VEGFC (Fig. 5A). To further investigate the correlation of TFAM and HIF1-α, we evaluated the expression of other HIF1-α targets across the cell lines (Fig. 5B) and, as a result, we could identify two clusters: one containing genes mostly up-regulated or unaltered in TFAM down cells (Fig. 5B, Cluster I) and other with genes mostly down-regulated (Fig. 5B, Cluster II). Additional investigation of the tumorigenic pathway involved showed that Cluster I genes were mainly involved in angiogenesis and invasion, and Cluster II genes in glycolysis, suggesting that TFAM expression can impact these pathways. The down regulation of glycolytic enzymes corroborates our previous findings that TFAM down melanoma cell lines indeed consumed less glucose (Fig. S3). Also, another metabolic enzyme, PHGDH, was found down-regulated in TFAM down cell lines (Fig. 5A), emphasizing a link between TFAM expression and the expression of genes regulating the glycolytic pathway.

Collectively, these results suggest that TFAM expression correlates with gene expression profiles linked to a variety of pro-tumorigenic pathways, namely angiogenesis, invasion and glycolytic metabolism. However, as we initially analyzed only a limited number of melanoma cell lines (n = 8), we next chose to further solidify our analysis by evaluating samples from metastatic melanoma tumours.

### TFAM correlates with metabolic gene expression changes in metastatic melanoma tumors

To expand our analysis of TFAM regulation of gene expression, we analyzed metastatic melanoma tumors using mRNA expression data from the TCGA database. To avoid bias in this analysis, we selected only metastatic melanoma samples harboring a mutation in the V600 BRAF codon (n = 127), as all the melanoma cell lines in our previous analysis also carried this mutation (Fig. S5A). Similar to our analysis in melanoma cell lines, we divided the melanoma tumors by TFAM expression, applying a cut-off of 25 and 75 percentiles to select samples with low TFAM expression (TFAM down, n = 32) and high TFAM expression (TFAM up, n = 32) (Fig. S5B).

**Figure 6:**
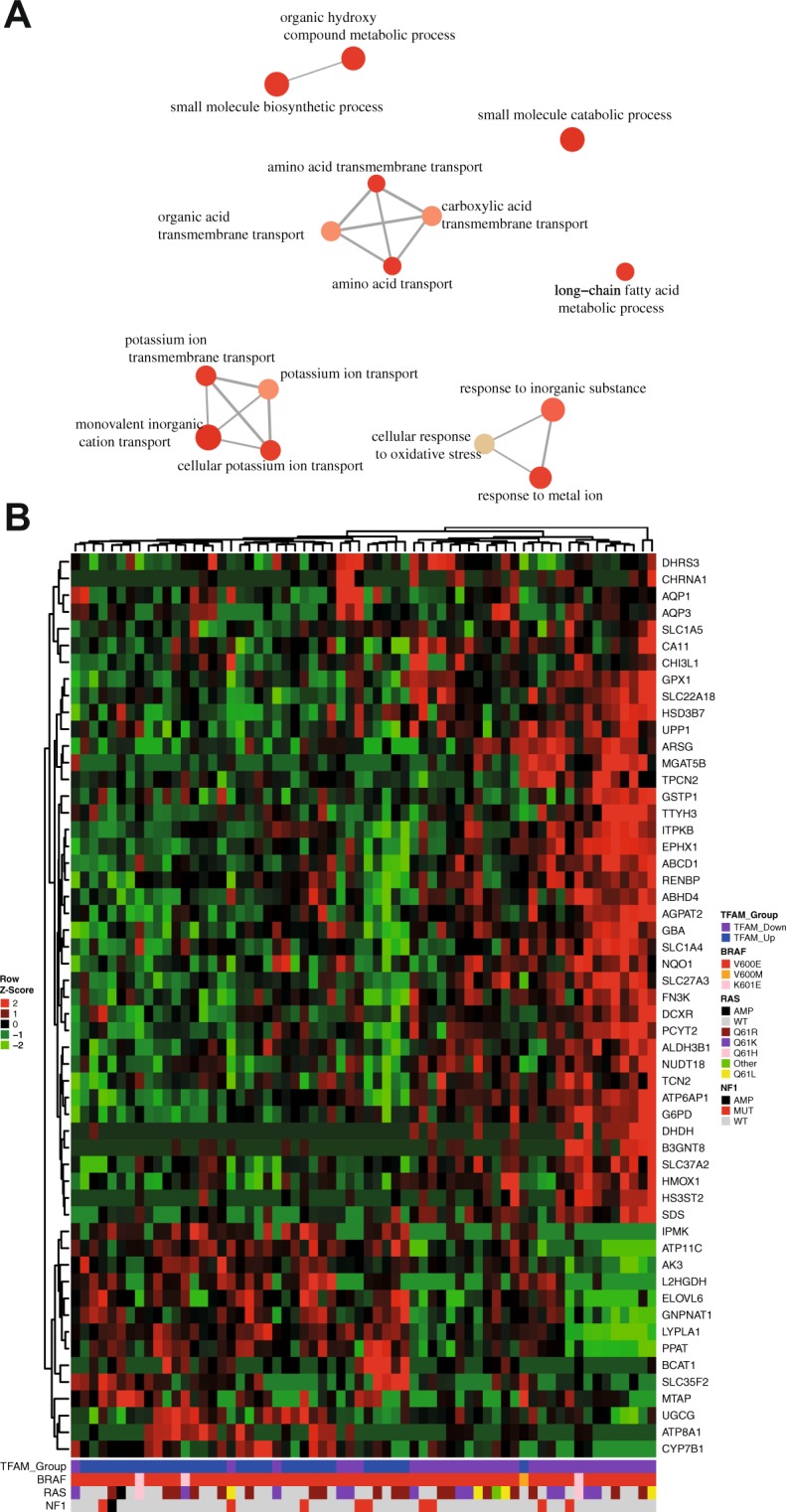
Differential expression analysis of metabolic enzymes between TFAM-Up and TFAM-Down in metastatic melanoma. (**A**) Gene ontology pathways enriched with the differentially expressed enzymes. Each circle represents gene pathways and correlated pathways are linked. The sizes of the circles are proportional to the enrichment analysis p-values; (**B**) Heatmap showing the top 54 genes differently expressed between TFAM up and down metastatic melanoma samples. Color bar at the bottom indicates the presence of mutations in BRAF, RAS and NF1 genes. AMP: Gene Amplification; WT: Wild-type; MUT: Mutated.

As the first portion of this study concerns metabolic alterations triggered by TFAM, we next used a list previously published by Possomato *et al*.^26^ to select differently expressed metabolic enzymes in our metastatic melanoma analysis and more broadly investigate changes in metabolic pathways. As a result, we found 100 DEGs, 68 up-regulated and 32 down-regulated in TFAM down samples (see Supplementary Table S2- DEGs Metabolic enzymes-TCGA). Enrichment pathway analysis with the DEGs revealed key pathways that could be regulated by TFAM, such as amino acid transport and cellular response to oxidative stress (Fig. 6A). Investigating the top differentially expressed genes, we noticed that the clustering was independent of mutations in the RAS and NF1 genes. Moreover, the samples seemed to cluster well based on the TFAM groups, indicating metabolic gene regulations cluster with TFAM expression (Fig. 6B).

The top 54 differentially expressed genes showed promising results, whereas GPX1 and GSTP1 gene expression was up-regulated in TFAM down samples. Both of these genes are major players in regulating oxidative stress (Fig. 6B). Also, genes related to amino acid pathways, SLC1A5 (ASCT2) and SLC1A4 (ASCT1) were both up-regulated in TFAM down samples, and the BCAT1 gene was down-regulated, suggesting that TFAM expression might be linked to amino acid metabolism. Additionally, we also found up-regulation of two metabolic biosynthesis key players, G6PD and ATP6AP1, in TFAM down samples, suggesting that TFAM levels influence the expression of genes coding for key components of metabolic pathways in melanoma.

### Decreased proliferation of TFAM down melanoma cell lines in low glutamine growth conditions

**Figure 7:**
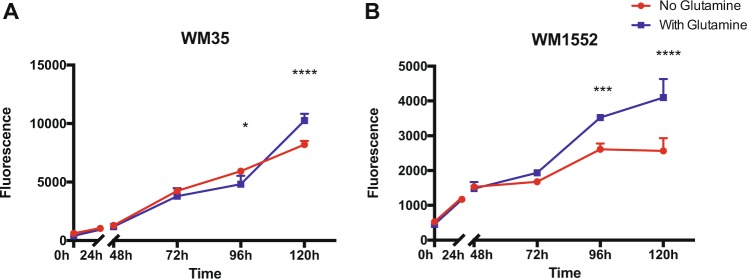
Proliferation assay of melanoma cell lines in different L-glutamine availability. TFAM-up melanoma cell line (WM35) had a similar growth under different L-glutamine availability (**A**) until 120 h (p < 0.0001), whereas cells cultured in media with glutamine grow more. On the other hand, there was a growth arrest in TFAM-down cell line (WM1552) in glutamine-free media at 96 h (p = 0.0004) that continued for 120 h (p < 0.0001). In all statistical analysis it was performed a two-way-ANOVA followed by Sidak’s multiple comparison test.

As observed before, gene expression analysis showed that ASCT2, mainly responsible for glutamine uptake, was up-regulated in TFAM down melanoma lines, suggesting that these cells might rely more on glutamine metabolism than TFAM up melanoma lines. To investigate this hypothesis, we measured the proliferation of WM35 (TFAM Up) and WM1552 (TFAM Down) in culture media with different L-glutamine availability. As a result, we found growth differences between TFAM up and down melanoma cell lines. TFAM up (WM35) growth was similar in both conditions (Fig. 7A). We observe growth arrest in TFAM down cells (WM1552) cultured in media without glutamine (Fig. 7B). We also performed this experiment in other melanoma cell lines and observed similar results (Fig. S6), supporting our hypothesis that TFAM down melanoma cell lines more readily require glutamine than lines with high TFAM expression.

### TFAM is associated with nuclear gene expression alterations in metastatic melanoma tumors

**Figure 8:**
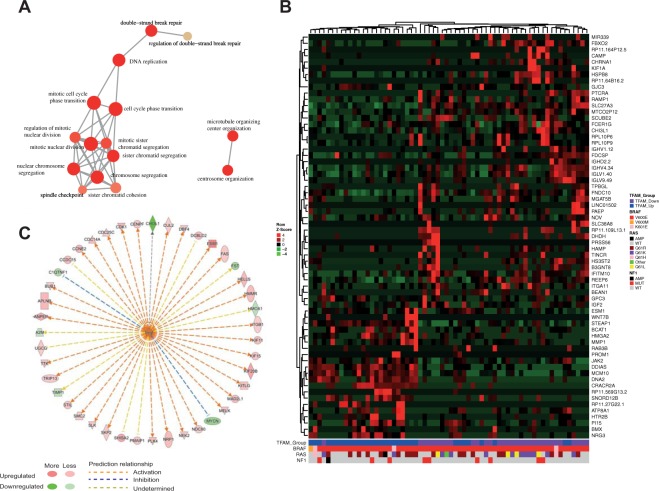
Differential expression analysis between TFAM-Up and TFAM-Down metastatic melanoma tumors from TCGA. (**A**) Gene ontology pathways enriched with the DEGs. Each circle represents gene pathways and correlated pathways are linked. The sizes of the circles are proportional to the enrichment analysis p-values; (**B**) Heatmap showing the top 65 genes differently expressed between the groups. Color bar at the bottom indicates the presence of mutations in BRAF, RAS and NF1 genes; (**C**) Upstream analysis plot representing the VEGF gene family (center) and its targets that were differently expressed in the analysis. Red circles indicate higher expression and green circles lower expression of the respective gene, as for the arrows, orange arrows indicate that VEGF was predicted to activate the gene and the blue arrow indicate that VEGF was predicted to inhibited. AMP: Gene Amplification; WT: Wild-type; MUT: Mutated.

In the previous analysis in melanoma cell lines (Fig. 5), we also found gene expression profiles linked to angiogenesis and invasion pathways. To further explore that, we performed a global gene expression analysis between TFAM up and down groups from TCGA Metastatic Melanoma samples. As a result, we found a total of 796 DEGs (FDR < 0.05), in which 385 were up-regulated and 411 were down-regulated in TFAM down samples (see Supplementary Table S2- DEGs TCGA). Gene ontology analysis of these genes revealed that many biological processes associated with cell cycle and DNA repair were enriched (Fig. 8A). Additionally, when we plotted the top 65 DEGs (Fig. 8B), we noticed that sample clustering was independent of mutations in the RAS (KRAS, NRAS, and HRAS) and NF1 genes, which have been described to modify gene expression patterns in melanoma, avoiding expression bias that might be caused by those genes. Similar to our data from melanoma cell lines (Fig. 5), we observed some VEGF targets in the top 65 DEGs, such as CHI3L1, KLF1 and ESM1 (Fig. 8C). We then performed an upstream regulator analysis using Ingenuity Pathway Analysis software and collectively noted that the VEGF gene family is a predicted upstream regulator of the expression profiles of TFAM down melanomas (Activation Z-score = 3.838, p-value = 7.71E-10). These data suggest a dynamic role of TFAM regulating diverse pathways, such as cell cycle and DNA repair, and they reinforce the inverse correlation between TFAM expression and angiogenesis pathway genes via VEGF modulation.

### TFAM correlation with invasion and mitochondrial biogenesis signature

As previous findings in both melanoma cell lines and TCGA data indicate that TFAM expression correlates with genes involved in invasion and proliferation (Figs 5B and 8C), we performed a Gene Set Variation Analysis (GSVA)^27^, from both datasets, aiming to calculate enrichment scores (ES) for invasion and proliferation signatures. After ES calculations, we performed a correlation analysis searching for an association of TFAM expression levels with each signature for every melanoma sample. Additionally, as TFAM plays a major role in mtDNA transcription, we also evaluated the mitochondrial biogenesis signature.

**Figure 9:**
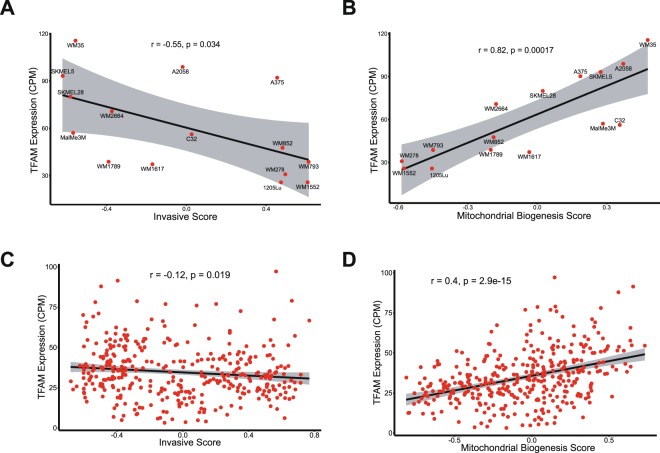
Correlation of TFAM expression with invasive and mitochondrial biogenesis signatures. Correlation analysis showed a negative correlation of TFAM expression with invasive signature in both melanoma cell lines (r = −0.55; p = 0.034) (**A**) and TCGA metastatic melanoma samples (r = −0.12; p = 0.019) (**C**). TFAM expression was also positive correlated with mitochondrial biogenesis in both cell lines (r = 0.82; p = 0.00017) (**B**) and TCGA data (r = 0.4; p = 2.9 × 10^−15^) (**D**).

The analysis showed a negative correlation of TFAM expression with invasive signature (Fig. 9A) and a positive correlation with mitochondrial biogenesis (Fig. 9B) in melanoma cell lines. Accordingly, a similar result was found in metastatic melanoma TCGA data (Fig. 9C,D). We did not find any correlation of TFAM with proliferative signature in either cell lines or the TCGA dataset (Fig. S7). These results support previous findings in gene expression analysis, as low TFAM expression seems to trigger invasion genes. Also, these data indicate that low TFAM expression largely correlates with an impaired mitochondrial biogenesis phenotype.

## Discussion

In this study, we undertook several novel approaches to investigate the relationship between mtDNA alterations and TFAM expression to energetic metabolism in melanoma cell lines. To expand our findings, we then investigated alterations in nuclear genes and metabolic enzyme expression triggered by TFAM levels. We reasoned that the impact of mtDNA alterations in melanoma would be informative despite lack of association with melanoma stage. Indeed, energetic metabolic analysis showed a positive correlation of low mtDNAcn with less glucose consumption and lower ATP production (Figs 3 and 4). The lower glucose consumption was later corroborated by gene expression analysis, which revealed down-regulation or little effect on glycolytic enzymes in TFAM down cell lines (Fig. 5B). A previous study using cancer cell lines lacking mtDNA showed similar results in terms of ATP production, but opposite findings regarding glucose consumption and lactate production^25^. As described by the Warburg effect, cancer cells consume higher amounts of glucose to compensate for OXPHOS deficiency^7^. However, many studies have shown that tumor metabolism is highly heterogeneous and can rely on alternative metabolic routes^8^.

We further evaluated signalling changes using gene expression data. Our expanded analysis focuses only on TFAM, as mtDNAcn data is not widely available in public datasets. Although TFAM depletion may not always indicate mtDNA instability, recent findings have shown that TFAM silencing impairs mitochondrial DNA packing^20,28^, leading to dispersed nucleoid formation and mtDNA released to the cytosol. Indeed, DNA repair pathways were enriched in the gene ontology analysis (Fig. 8A) and we also observed a strong correlation between TFAM expression and mitochondrial biogenesis signature (Fig. 9B,D), reinforcing the role of TFAM in maintaining mtDNA and mitochondrial stability.

Additionally, it was previously described that cancer cells with mitochondrial dysfunction rely mostly on α-ketoglutarate to supply bioenergetic requirements^10^. Alpha-ketoglutarate can be generated from glutamine metabolism, as an alternative metabolic source of carbon^29,30^. Our gene expression analysis in metastatic melanoma tumors with different TFAM levels (Fig. 6) showed an enrichment of amino acid transport pathways and an up-regulation of SLC1A5 (ASCT2), SLC1A4 (ASCT1) and ATP6AP1. SLC1A5 and SLC1A4 are both amino acid transporters, the former mostly responsible for glutamine influx and the latter for serine^31,32^. ATP6AP1 is a subunit of the the ATPase complex V, responsible for sensing amino acids levels and activating mTORC1 signalling, which controls protein synthesis^33^. Likewise, our proliferation analysis suggests that low TFAM melanoma cell lines may depend on glutamine availability to maintain optimum cellular growth (Fig. 7). We also observed an up-regulation of G6PD, an enzyme that acts on the first step of the pentose phosphate pathway, an extremely important pathway for cancer cells that generates the phosphate required for nucleic acid synthesis^34^. These data suggest that to bypass the down-regulation of glycolysis, TFAM can regulate the energetic metabolism towards amino acid pathways, especially glutamine, to maintain the metabolic requirements of the cancer cells.

Similar to our findings, Yang *et al*.^35^ observed that highly invasive ovarian cancer cells are highly dependent of glutamine. On the other hand, Baenke *et al*.^36^ showed that Vemurafenib resistant melanoma cells also rely more on glutamine metabolism. However they displayed an increased mitochondrial biogenesis, in contrast to our finding, whereas TFAM depletion seems to both increase the dependence on glutamine pathways and decrease the signature of mitochondrial biogenesis.

Moreover, the role of TFAM in the regulation of oxidative stress remains unclear in our analysis. Previous studies have shown that depletion of TFAM triggers ROS production *in vivo*^18,19^, but our study failed to correlate hydrogen peroxide and TFAM expression (Supplementary Fig. S1). However, metastatic tumor analysis revealed an up-regulation of GPX1 and GSTP1, which act on the conversion of hydrogen peroxide to water^37^. This suggests a response to high levels of ROS in TFAM down samples (Fig. 6). As melanoma tumor analysis was performed in a larger number of samples, we believe that the limited number of melanoma cell lines might compromise any correlation of TFAM and hydrogen peroxide in our data.

Our analysis also revealed two targets of HIF1-α up-regulated in TFAM down cell lines: LOX, responsible for invasive properties of hypoxia cancer cells mainly through mechanisms of cell adhesion^38^ and VEGFC, which promotes lymphangiogenesis^39^ and was previously associated with metastasis^40,41^. HIF1-α stabilization is the major response mechanism to hypoxia, and has been described in many cancer types. It can trigger expression of pro-tumorigenic genes, such the ones that we observed to be up-regulated in our TFAM down melanoma cell lines^38^.

Furthermore, gene expression analysis in both melanoma cell lines and metastatic melanoma tumors suggested that the depletion of TFAM triggers angiogenesis via VEGF (Figs 5 and 8) and is correlated with an invasion gene signature (Fig. 9). Indeed, previous studies have reported tumor growth in an intestinal cancer model^18^, emphasizing a pro-tumorigenic role of TFAM depletion. However, none of these studies have evaluated VEGF expression. Interestingly, a study with non-small cell lung carcinoma suggested an anti-tumorigenic role of TFAM, whereas silencing of this gene triggered cell cycle arrest, inhibiting cancer cell proliferation and migration^42^. Gene ontology analysis of our data also showed many cell cycle pathways enriched. Additionally, recent studies have suggested multiple function for TFAM once this protein is present in tumor cells that lack mtDNA^25,43^. Due to high heterogeneity among cancer types, we believe that only an *in vivo* analysis using a melanoma model can confirm the tumorigenic role of TFAM in melanomagenesis.

In conclusion, our study employs multiple bioinformatic and *in vitro* approaches to evaluate the role of TFAM in melanoma cell lines and metastatic melanoma tumors. We have found that mtDNAcn/TFAM is correlated with glucose consumption and ATP production, and gene expression analysis suggests that TFAM down-regulation may shift cells and tumors from dependence on glucose toward glutamine metabolism, in order to supply an alternative source of carbon independent of glucose to maintain the metabolic needs of melanoma cells. Additionally, our analysis supports a pro-tumorigenic signaling role for TFAM, which has been previously suggested in other tumor types^18,19^, and we provide new data supporting that low TFAM expression drives invasion via VEGF and the expression of a more invasive gene expression signature. Our findings therefore expand the understanding of TFAM in cancer, and provide new insight into its diverse roles in shaping melanoma metabolism, growth, and invasion.

## Methods

### Cell culture

We used a set of melanoma cell lines that individually represent the stages of melanoma progress: WM35, WM1552C and WM1789 representing the RGP; WM278, WM902, WM793, representing the VGP; and 1205 LU, WM1617 and WM9, representing metastatic melanomas. The pairs WM278/WM1617 and WM793/1205 LU were established from the same patient. The WM melanoma cell lines were cultivated as previously described^44^. Additionally, we used melanocytes previously isolated from neonatal foreskin (FM308) and maintained according to Sousa and Espreafico, 2008^45^ and Sousa *et al*.^45^. All the cell lines were kindly provided by Meenhard Herlyn (The Wistar Institute, Philadelphia, PA).

### Mitochondrial genome sequencing and analysis

DNA and RNA were isolated from the cell lines with the AllPrep DNA/RNA/miRNA Universal kit (Qiagen), following the manufactures protocol. The DNA was used for whole exome analysis as described previously^46^. Briefly, for the exome library preparation, we used the Nextera Exome Enrichment kit (Illumina) and then proceeded with 55-bp paired-end sequencing using the TruSeq SBS v5 Kit, in the Genome Analyzer IIx (GAIIx) Illumina platform. Sequencing.bcl basecall files were demultiplexed and formatted into.fastq files using CASAVA software (Illumina), followed by quality control in the FastQC software. Fastq files were then aligned using the BWA-MEM^47^, generating BAM files.

The BAM files were used in the Mitoseek software^48^, which extracts the reads mapped against the mtDNA, align them to the mitochondrial reference sequence rCRS^23^ and call variants.

### mtDNA copy number analysis

Mitochondrial genome content was evaluated as previously described at De Araujo *et al*.^46^. Briefly, we quantify mtDNAcn by qPCR, using Power SYBR Green Master Mix (Thermo Scientific) in a 7500 Fast Real-Time System (Applied Biosystems). We determined the relative copy number of mitochondrial DNA with set of primers specific for the mtDNA and the nuclear genome, as previously reported by Venegas *et al*.^49^.

### RNA-seq analysis

For the RNA-seq analysis, after integrity evaluation, 300 ng of RNA were used for library preparation with the TruSeq Stranded Total RNA LT Sample Prep kit with RiboZero (Illumina). The sequencing reaction proceeded as described before in the whole-exome sequencing: a 55-bp paired-end sequencing using the TruSeq SBS v5 Kit, in the Genome Analyzer IIx (GAIIx) Illumina platform. The software STAR was used to map the reads (GRCh38 -Gencode), count and annotate the transcripts^50^. Normalization and differential expression analysis were performed with edgeR package in R^51^. False discovery rate (FDR) was used to adjust p values. Genes with adjusted p value < 0.05 were considered differentially expressed. Volcanos plots and heatmaps were also generated in R.

### Public data analysis

To expand our melanoma cell lines panel, we used publicly available data generated elsewhere and deposited under the code GSE46818 at GEO^24^. The data consisted on a RNA-seq from seven melanoma cell lines (A375, A2058, c32, MalMe3M, SKMEL28, SKMEL5, WM2664) and one melanocyte (EBRAF-MC), also sequenced at the GAIIx (Illumina). The data was downloaded in sra file and converted to fastq using SRA toolkit^52^. Then, the fastq files were combined with the data that we have generated before and analyzed as we described.

Metastatic melanoma mRNA expression data from The Cancer Genome Atlas (TCGA) platform was downloaded through the Broad GDAC Firehose (https://gdac.broadinstitute.org/). Clinical information and mutational data on the BRAF, NRAS, KRAS and NF1 genes were retrieved using the cBioPortal^53^. The data was compiled in R and differential expression analysis was performed as mentioned before using edgeR^51^. Gene ontology enrichment analysis was performed with the clusterProfiler package, also in R^54^, and upstream regulators analysis with Ingenuity pathway analysis.

### Gene set variation analysis

Gene Set Variation Analysis (GSVA) was performed as previously established by Hänzelmann *et al*.^27^, using melanoma cell lines and metastatic melanoma TCGA normalized gene expression data. Invasive and proliferative melanoma gene expression signature were obtained from Widmer *et al*.^55^ and mitochondrial biogenesis signature from Zhang *et al*.^56^. Pearson’s correlation coefficient was used for the correlation analysis of each ES with TFAM.

### Hydrogen peroxide measurement

To measure hydrogen peroxide (H_2_O_2_) production, we used the Amplex Red kit (Amplex Red Hydrogen Peroxide/Proxidase Assay Kit (Invitrogen), according to the manufacturer’s instructions. Briefly, we prepared a Krebs-Ringer-phosphate solution with glucose (KRPG: 145 mM of NaCl, 5.7 mM of sodium phosphate, 4.86 mM of KCl, 0.54 mM of CaCl_2_, 1.22 mM of MgSO_4_, 5.5 mM of glucose, pH de 7.35), containing 50 µM of Amplex Red and 0,1 U/mL of peroxide hydrogen oxirredutase. We added 1 × 10^5^ cells to this solution and incubated for 10 min at 37 °C. The fluorescence was quantified for 60 min in the FLUOstar Omega plate reader (BMG Labtech). All the reactions were performed in triplicate. As a positive control, we treated the cells with 250 µM of t-butil (pro-oxidant agent) for 30 min.

### ATP measurement

Intracellular ATP measurements were performed using the CellTiter-Glo Assay (Promega), following the manufactures’ instructions. Briefly, we added 50 µl of the Celltiter-glo reagent in a well containing 5 × 10^3^ cells that were plated the night before. The cells were agitated for 2 min at 700 rpm to the lysate. After 10 min incubation, the fluorescence was read in the FLUOstar Omega plate reader (BMG Labtech). All the reactions were performed in triplicate and, we also treated the cells with 10 µM of oligomycin (ATP synthase blocker) for 30 min, as control of the reaction.

### Glucose consumption and lactate production rates

To measure the glucose consumption and the lactate production, 1 × 10^5^ cells were plated in triplicate for 6 days. In every 24 hours, we collected the supernatants and counted the number of cells per well. After the 6^th^ day, concentrations of glucose and lactate were measured in the YSI 2700 Select Biochemestry Analyzer.

The glucose consumption rate (qGlc) and lactate production rate (qLac) were calculated as previously described^57^. The rates encompass the amount of metabolite consumed or produced, taking into account the number of cells per day. The rates were calculated during the exponential growth phase of each cell line.

### Proliferation assay

Proliferation assay was performed using the CyQuant Direct Cell Kit (Invitrogen), according to the manufactures’ instructions. Briefly, 5 × 10^3^ cells were plated in triplicate and cultured in RPMI media 1640 (VitroCell) in two different conditions: culture media without L-glutamine and supplemented with glutamine (2 mM). In every 24 hours, we removed the media, added 100 µl of CyQuant reagent and incubated for one hour at 37 °C. After incubation, fluorescence was read in the FLUOstar Omega plate reader (BMG Labtech).

### Statistical analysis

Statistical analysis and scatter plots generations were performed in R using the package R commander^58^ and bar plots of the metabolic assays were generated in GraphPad Prism 6. The level of statistical significance was established at p < 0.05. The statistical tests that were applied are described in their respective results.

## Electronic supplementary material

Supplementary figures

Supplementary table 1

Supplementary table 2
