# Supplementary material for: Mitochondrial transcription factor A (TFAM) shapes metabolic and invasion gene signatures in melanoma
Source: Sci Rep. 2018 Sep 21;8:14190. doi: 10.1038/s41598-018-31170-6 (PMC6155108; doi:10.1038/s41598-018-31170-6)
Supplement: Supplementary file 1 — Supplementary figures [file 41598_2018_31170_MOESM1_ESM.pdf]

Mitochondrial transcription factor A (TFAM) shapes metabolic and invasion gene signatures in melanoma

Araujo LF, Siena ADD, Placa JR, Brotto DB, Barros II, Muys BR, Biagi Junior CAO, Peronni KC, Sousa JF, Molfetta GA, West LC, West AP, Leopoldino AM, Espreafico EM, Silva WA Jr

Supplementary Figures

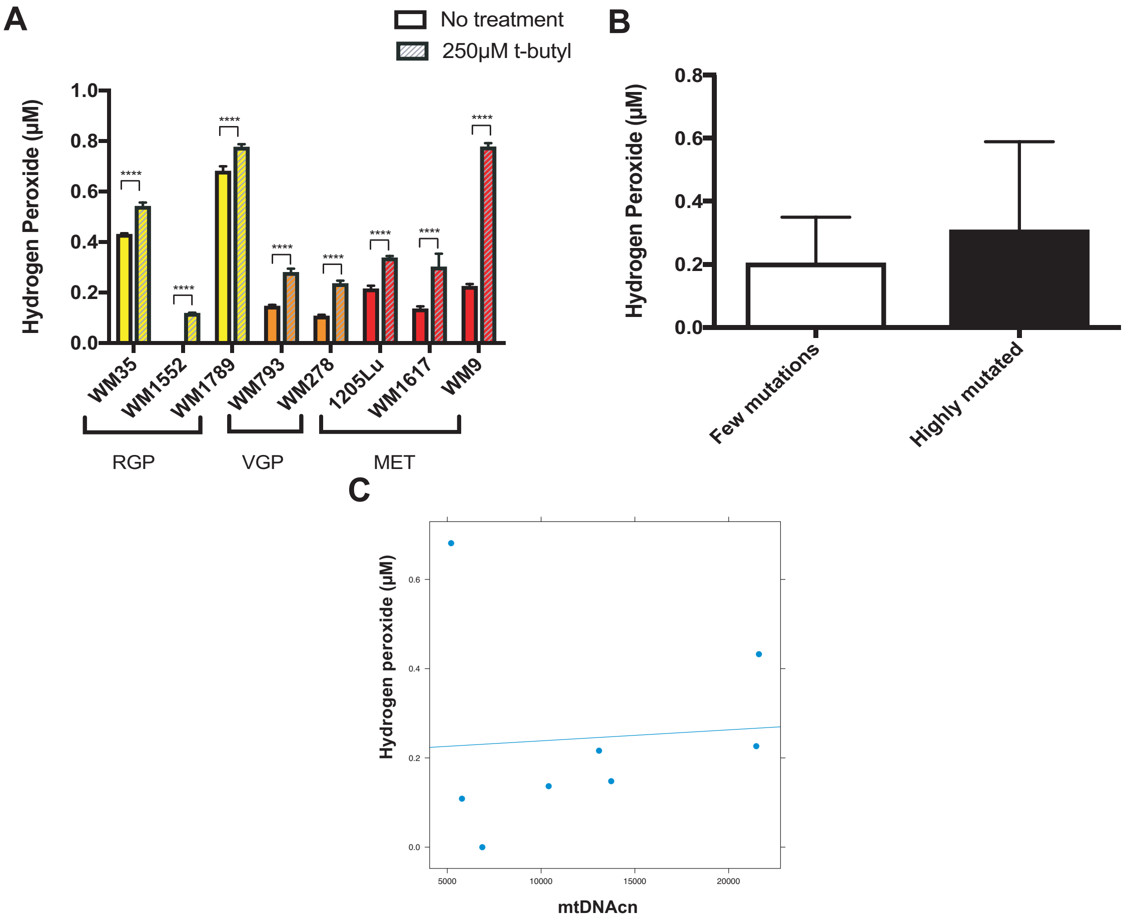

Figure S1: Hydrogen peroxide measurement in the melanoma cell lines. (A) Hydrogen peroxide levels in the melanoma cell lines. The t-butyl treatment was used as a control of the reaction. (B) There was no difference in the Hydrogen peroxide levels between the differetially mtDNA mutated clusters (p=0.2387, Student t test). (C) We also didn't find any correlation between hydrogen peroxide levels and mtDNAcn (r=0.0745, p=0.5696, Pearson Correlation). RGP: Radial Growth Phase; VGP: Vertical Growth Phase; MET: Metastatic Melanoma; mtDNAcn: Mitochondrial DNA copy number. Hydrogen peroxide levels were evaluated in the following melanoma cell lines: WM35, WM1552, WM1789, WM793, WM278, 1205Lu, WM1617 and WM9.

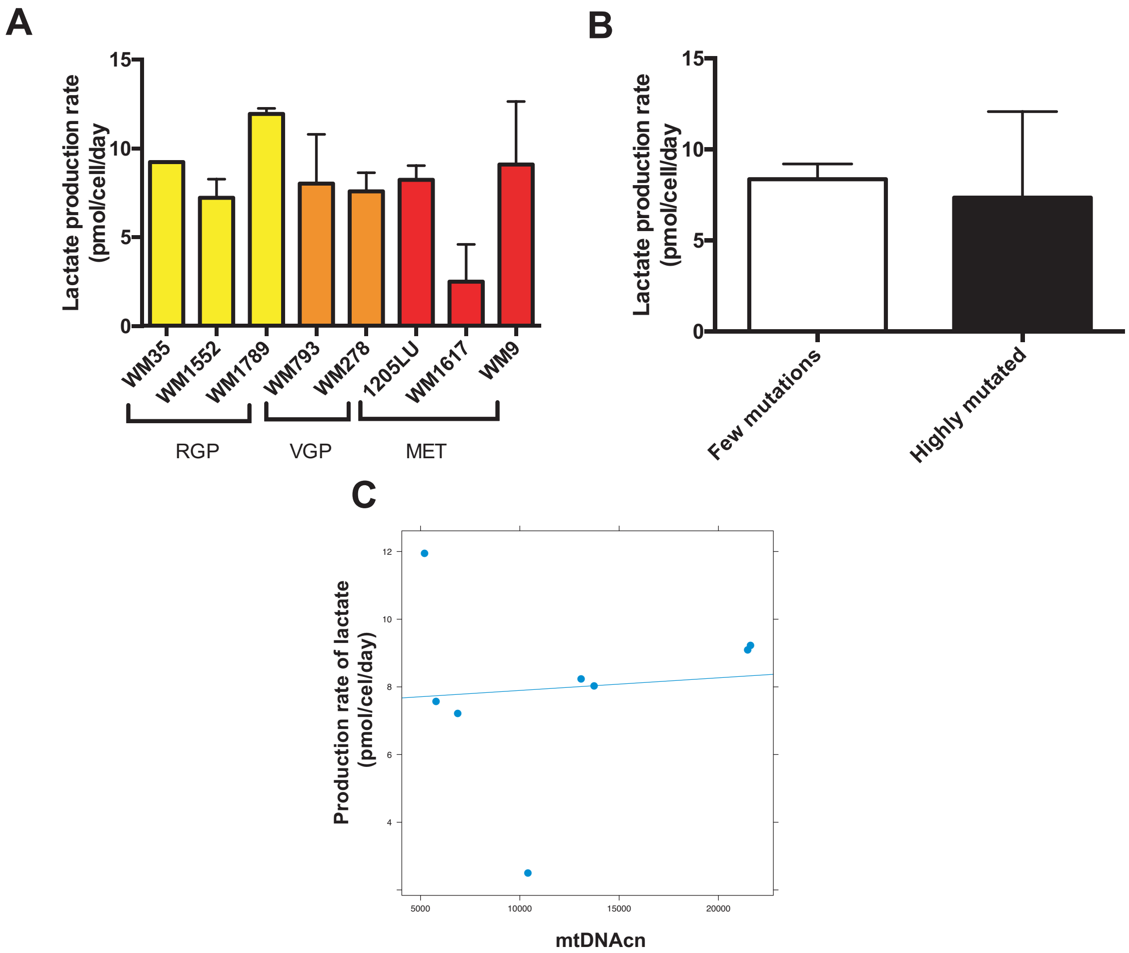

Figure S2: Lactate production rate in the melanoma cell lines. (A) Lactate production rate (qLac) per melanoma cell lines.. (B) There was no difference in the qLac between the differetially mtDNA mutated clusters (p=0.6373, Student t test); (C) We also didn't find any correlation between qLac rates and mtDNAcn (r=0.0917, p= 0.4144, Pearson Correlation). RGP: Radial Growth Phase; VGP: Vertical Growth Phase; MET: Metastatic Melanoma; mtDNAcn: Mitochondrial DNA copy number. Lactate production rates were evaluated in the following melanoma cell lines: WM35, WM1552, WM1789, WM793, WM278, 1205Lu, WM1617 and WM9.

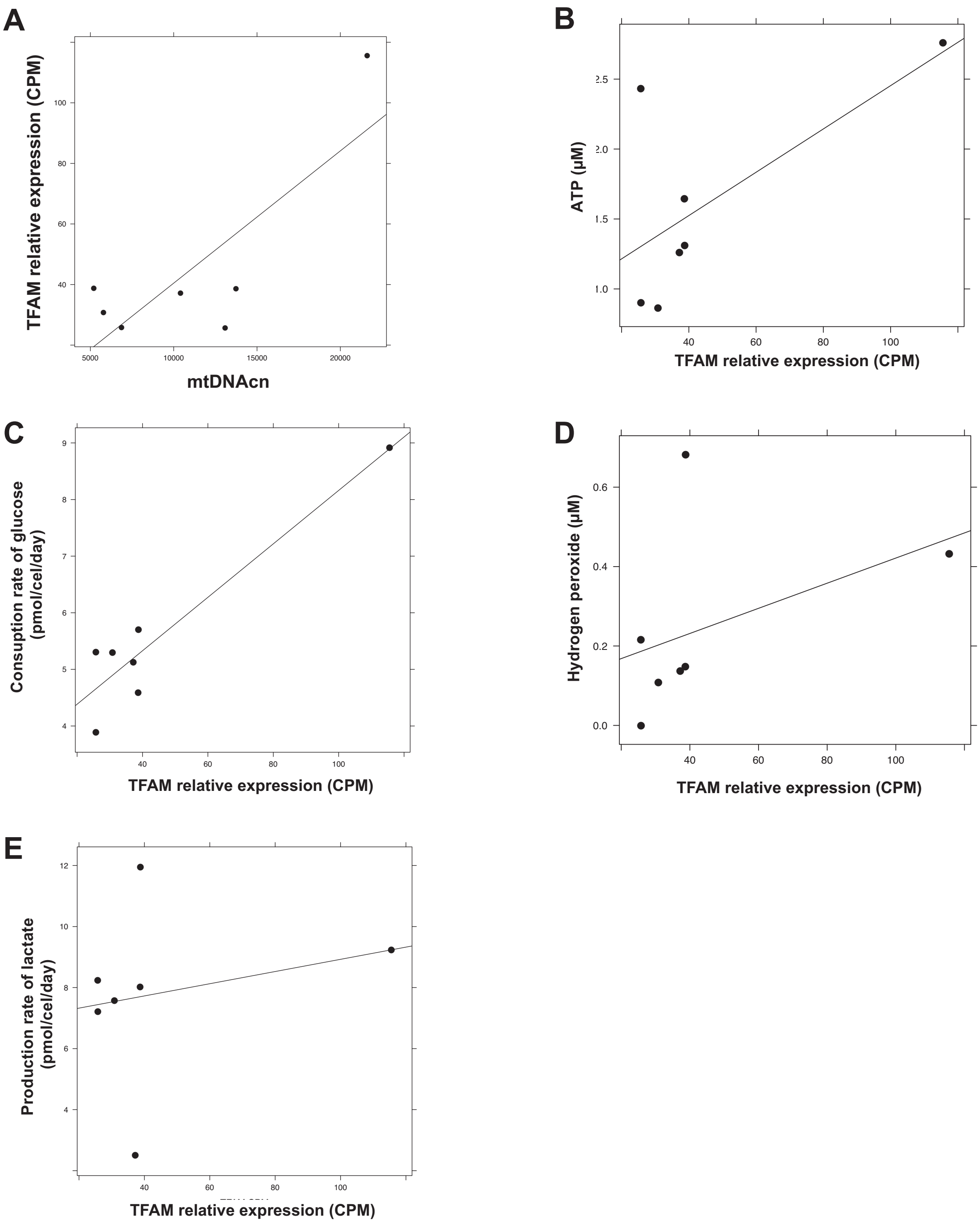

Figure S3: Correlation analysis of TFAM expression and mtDNAcn and metabolic parameters in the melanoma cell lines. No correlation was found between TFAM expression and mtDNAcn (rho= 0.2142; p=0.3308, Spearman's correlation) (A), ATP production (rho= 0.39; p=0.1978, Spearman's correlation) (B), glucose consuption (rho= 0.46; p=0.2667, Spearman's correlation) (C), hydrogen peroxyde production (rho= 0.60; p=0.0833, Spearman's correlation) (D) and lactate production (rho= 0.50; p=0.1333, Spearman's correlation) (E). CPM: Counts per Million. RNA-seq analysis was not performed in WM9 and WM902. The dots at figure B-E represents in the following melanoma cell lines: WM35, WM1552, WM1789, WM793, WM278, 1205Lu and WM1617.

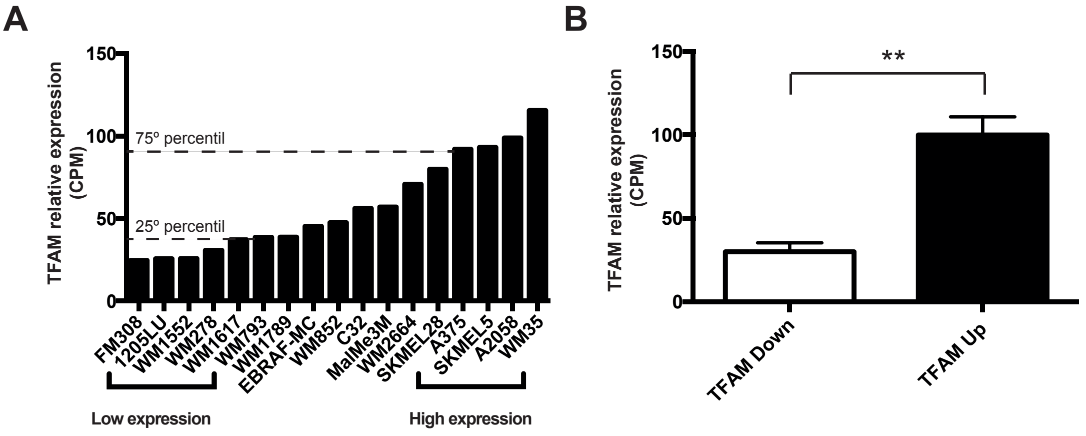

Figure S4: TFAM expression in the melanoma cell lines. (A) TFAM expression across the melanoma cell lines; (B) Differential expression between the melanoma cell lines with high and low TFAM expression (p= 0,0001383, student t test).

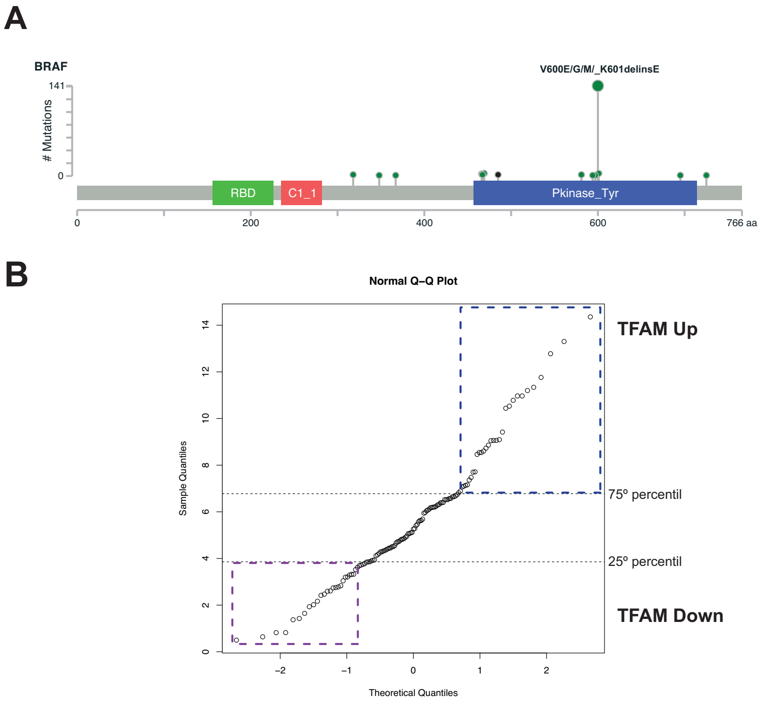

Figure S5: TFAM expression in the metastatic melanoma tumors. (A) Lolliplot showing the BRAF genes and the mutations found in melanoma samples, including metastatic and primary. To avoid bias, in our analysis we only used metastatic samples; (B) Quantil-quantil plot with the TFAM expression across all the metastatic melanoma samples harboring the BRAF^{V600E} mutation. For our analysis, we selected TFAM down and TFAM up samples based on the percentiles.

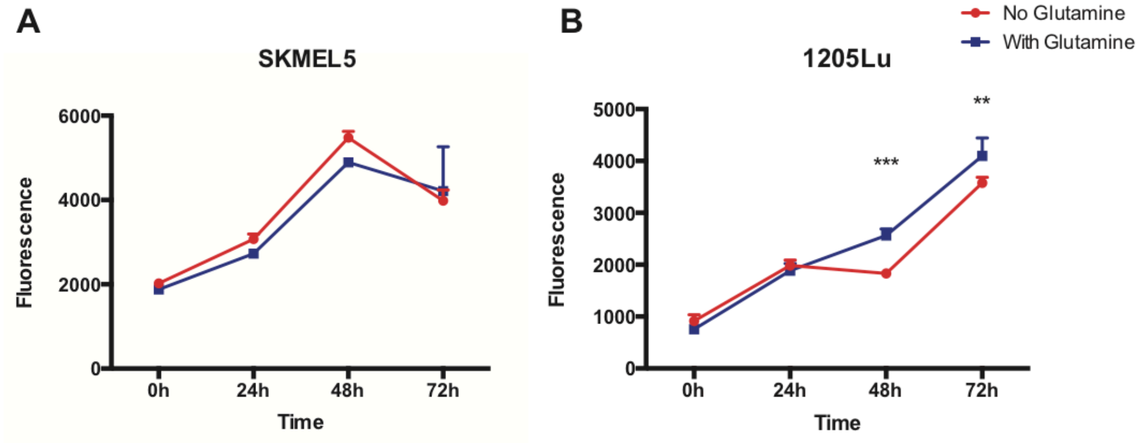

Figure S6: Proliferation assay of melanoma cell lines in different L-glutamine availability. There was no growth change in TFAM-up melanoma cell line (SKMEL5) in different L-glutamine availability (A). However, there was a growth arrest in TFAM-down cell line (1205Lu) in glutamine-free media at 48h ($p=0.0001$) that continued until 72h ($p=0.0037$) (B). In all statistical analysis it was performed a two-way-ANOVA followed by Sidak's multiple comparison test.

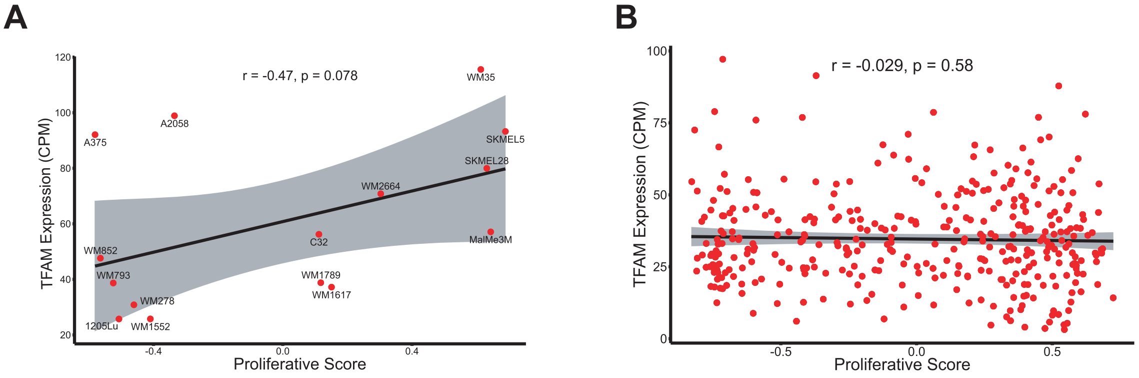

Figure S7: Gene Set Variation Analysis (GSVA) of TFAM expression with proliferative signature. No correlation was found between TFAM expression and proliferative signature in both melanoma cell (A) and TCGA metastatic melanoma samples (B).
